# Association of Type 2 Deiodinase Thr92Ala Polymorphism with Pediatric Obesity in Japanese Children: A Case-Control Study

**DOI:** 10.3390/children9101421

**Published:** 2022-09-20

**Authors:** Takeshi Ota, Jun Mori, Yasuhiro Kawabe, Hidechika Morimoto, Shota Fukuhara, Kazuki Kodo, Satoru Sugimoto, Kitaro Kosaka, Hisakazu Nakajima, Hajime Hosoi

**Affiliations:** Department of Pediatrics, Graduate School of Medical Science, Kyoto Prefectural University of Medicine, Kyoto 602-8566, Japan

**Keywords:** pediatric obesity, brown adipose tissue, type 2 deiodinase, uncoupling protein 1, β3 adrenergic receptor

## Abstract

Genetic factors play critical roles in the onset and progression of obesity. Brown adipose tissue (BAT) activity is also critical for adiposity. The objective of this study was to evaluate the prevalence and effects of BAT gene polymorphisms in pediatric obesity. This case-control study included 270 non-obese and 86 obese children. All participants underwent genotyping for type 2 deiodinase (DIO2) Thr92Ala (rs225014). The prevalence of the homozygous Ala/Ala allele of the DIO2 gene in the obese group was 15.1% versus 6.3% in the non-obese group, resulting in an odds ratio (OR) of 3.393 (*p* = 0.003). The results of this study indicate that the homozygous Ala/Ala allele of the DIO2 gene is associated with an increased risk of pediatric obesity and suggest that pediatric obesity might be suitable for assessing the association with gene polymorphisms related to BAT, especially DIO2 Thr92Ala.

## 1. Introduction

Obesity is a complex medical disease caused by multiple factors, including genetic susceptibility, the influence of the environment, and lifestyle practices. It is associated with an increased risk of various diseases, including cardiovascular disease, type 2 diabetes mellitus, and cancer [1]. Obesity is increasing globally, even in children. Pediatric obesity tends to be followed by continued obesity through adulthood. Thus, the epidemic of pediatric obesity spreading globally is considered one of the most important health issues prevalent today. Genetic factors are involved in 40–70% of cases of obesity [2], and various single-nucleotide polymorphisms (SNPs) are associated with pediatric obesity [3]. Hence, there is a crucial need to evaluate the genetic background associated with pediatric obesity.

Brown adipose tissue (BAT) has recently been identified as a novel target for obesity. Obesity is caused by a state of surplus energy, in which energy intake exceeds energy expenditure, leading to storage of the surplus. BAT increases energy expenditure through non-shivering thermogenesis. Some studies have reported that gene polymorphisms related to BAT are involved in the pathogenesis of obesity during adulthood [4,5,6,7]. However, only a few studies have focused on the association between gene polymorphisms related to BAT and childhood obesity. While BAT exists in adulthood, it is more abundant during childhood. Therefore, BAT activity is likely to be critical for adiposity in children. Enhancing BAT may be an attractive strategy to control pediatric obesity [8,9,10]. Based on reviewing this information, we can conclude that the effects of gene polymorphisms associated with BAT may be greater in childhood obesity than in adulthood.

Type 2 deiodinase (DIO2) converts thyroxine to triiodothyronine (T3), which is biologically active. Thyroid hormone (TH) stimulates thermogenesis through induction of mitochondrial uncoupling protein 1 (UCP1) in BAT. The thermogenic effect of BAT in response to TH is caused by synergistic interaction with the sympathetic nervous system (SNS) through the β3 adrenergic receptor (β3AR). TH receptors, both α- and β-subunits, are expressed in BAT. Synergism between TH and SNS causes an effect via TRα, and the transcription of UCP1 is upregulated by TRβ [11]. In addition, increased tissue levels of T3 amplify the SNS effects, including UCP1 gene transcription.

The present study focused on the DIO2, UCP1, and β3AR genes, which are key molecules in BAT thermogenesis. Some studies have reported that these genes are associated with the pathogenesis of adulthood obesity [4,5,6,7]. However, other studies revealed that these genes have no impact on the pathogenesis of adulthood obesity [12,13,14]. The reasons for these inconsistent results and the impact of these genes on pediatric obesity remain unknown. Therefore, we examined the effects of DIO2 Thr92Ala (rs225014), UCP1-3826 A/G (rs1800592), and β3AR Trp64Arg (rs4994) polymorphisms on obesity in Japanese children.

## 2. Materials and Methods

### 2.1. Subjects

This study was approved by the Institutional Review Board (IRB) of Kyoto Prefectural University of Medicine (IRB number: RBMR-G-71-7). BMI% or z-score is widely used to assess obesity worldwide. However, in this study, we classified individuals with the percentage of overweight (POW) ≥ 20% as obese, according to Japanese guidelines [15]. POW, which is the modified weight-for-height method, is widely used as a surrogate marker of childhood obesity in Japan [16]. POW is calculated using the following formula:

POW (%) = 100 × (measured weight − standard weight)/standard weight

**Figure 1:**
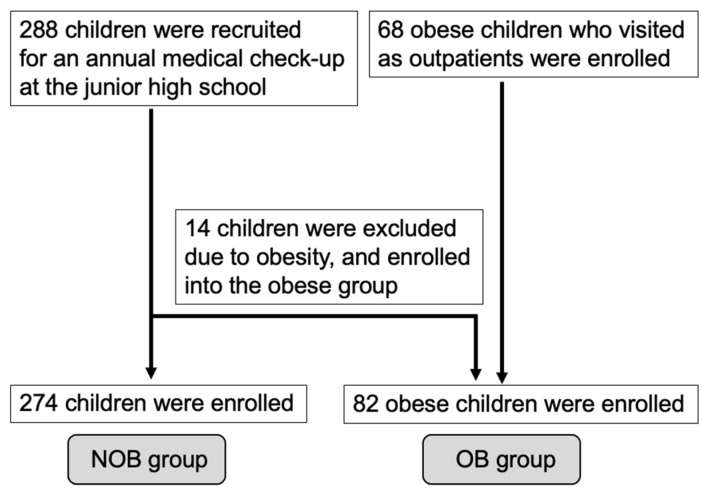
Flowchart showing the process for data selection.

The Japanese standard weight is expressed in terms of the age- and sex-specific weight for height, based on data from the Annual Report of School Health Statistics 2000, collated by the Ministry of Education, Culture, Sports, Science, and Technology, Japan. POW is reported to be a more appropriate method than BMI% for school-age children, as BMI overestimates tall and short children as being overweight and underweight, respectively [17]. A POW of 20% is equivalent to approximately the 90th BMI% of children with average height and weight, and the criteria for obesity are defined as POW ≥ 20% (≥120% of the standard weight) [17]. To collect data on non-obese children (NOB), study participants were recruited during an annual medical check-up at a junior high school in Kyoto. The NOB group did not include children with any underlying conditions. Informed consent was obtained from all 288 children and their parents. Fourteen children were excluded from the NOB group and included in the obese children (OB) group because of high POW. Finally, 274 children were enrolled in the NOB group. The median (range) age was 13.5 years (12.1–15.2). In the OB group, 68 obese children who visited our outpatient clinic were included. Informed written consent was obtained from the parents of these children. Finally, 82 children were enrolled in the OB group (including the 14 children recruited from the junior high school) (Figure 1). The median (range) age was 11.1 years (4.6–17.5) (Table 1). The presence or absence of obesity was also determined using the BMI%, and the same analysis was performed (Table 1).

### 2.2. Genotyping

Genomic DNA was extracted from blood leukocytes using a genomic DNA separation kit (DnaQuick II, DS Pharma Biomedical, Osaka, Japan). We analyzed the following polymorphisms in three genes: DIO2 Thr92Ala (rs225014), UCP1-3826 A/G (rs1800592), and β3AR Trp64Arg (rs4994). Each gene was detected via polymerase chain reaction (PCR) using the following forward and reverse primers: DIO2 forward primer = 5′-GGTACCATTGCCACTGTTGTCA-3′, DIO2 reverse primer = 5′-GTCAGGTGAAATTGGGTGAGGAT-3′, UCP1 forward primer = 5′-CCAGTGGTGGCTAATGAGAGAA-3′, UCP1 reverse primer = 5′-GCACAAAGAAGAAGCAGAGAGG-3′, β3AR forward primer = 5′-CGCCCAATACCGCCAACAC-3′, β3AR reverse primer = 5′-CCACCAGGAGTCCCATCACC-3′.

Genotyping was performed using the ABI 7500 Fast Real-time PCR System (Applied Biosystems, Foster City, CA, USA), TaqMan^®^ Genotyping PCR Master Mix (Thermo Fisher Scientific, Waltham, MA, USA), and TaqMan^®^ SNP Genotyping Assay (Thermo Fisher Scientific).

### 2.3. Biochemical Analysis

The following clinical data were collected for each test subject: sex, age at first visit, height, weight, POW, BMI, serum total cholesterol (TC), high-density lipoprotein cholesterol (HDLC), low-density lipoprotein cholesterol (LDLC), non-HDLC, and hemoglobin A1c (HbA1c).

### 2.4. Statistical Analysis

Clinical and laboratory data were summarized as the median (25th–75th) for continuous variables and number (%) for categorical variables and compared between groups stratified according to obesity status (NOB, OB) and genotype prevalence using the Mann–Whitney U test or Fisher’s exact test. Association between the DIO2/UCP1/β3AR genotypes and obesity was analyzed using cross-tabulation and Fisher’s exact test. Univariate and multivariate regression analyses were performed to evaluate the contribution of genetic polymorphisms to obesity. For multivariate analysis, each genotype in each gene was included as an independent variable: DIO2 Thr/Thr, DIO2 Thr/Ala, DIO2 Ala/Ala, UCP1 AA, UCP1 AG, UCP1 GG, β3AR Trp/Trp, β3AR Trp/Arg, and β3AR Arg/Arg. In multivariate analysis, a backward stepwise selection method was applied, and the best-fit model was determined according to the Akaike information criterion. In this exploratory study, sample size was not statistically determined, and multiplicity adjustment was not considered in the statistical analysis. Statistical significance was set at *p* < 0.05. All statistical analyses were performed using SPSS version 26.0 (IBM Corp., Armonk, NY, USA).

## 3. Results

### 3.1. Clinical Characteristics of the Subjects

The clinical characteristics of the NOB and OB groups classified according to POW are shown in Table 1. The median age and height of the NOB group were higher than those of the OB group. For lipid profiles, TC, LDLC, and non-HDLC levels in the OB group were significantly higher than those in the NOB group, and HDLC levels in the NOB group were significantly higher than those in the OB group. For glucose metabolism parameters, HbA1c levels in the OB group were significantly higher compared to those of the NOB group.

### 3.2. Genotype Distributions of SNPs

The genotype distributions of DIO2 Thr92Ala (rs225014), UCP1-3826 A/G (rs1800592), and β3AR Trp64Arg (rs4994) in the NOB and OB groups are shown in Table 2. The prevalence of the homozygous Ala allele of DIO2 in the OB group was significantly higher than that in the NOB group for the Thr/Thr and Ala/Ala genotypes (*p* = 0.004). The genotype distributions of UCP1-3826 A/G (rs1800592) and β3AR Trp64Arg (rs4994) did not significantly differ between the obese and non-obese groups (*p* = 0.123 and 0.716, respectively). The outcomes of the uni- and multivariate analyses with respect to obesity are summarized in Table 3. DIO2 Ala/Ala was associated with obesity, with an odds ratio (OR) of 4.036 (95% CI 1.785–9.124, *p* = 0.003) via univariate analysis. In contrast, ORs of UCP1-3826 G/G and β3AR Arg64Arg were 1.757 (95% CI, 0.893–3.458, *p* = 0.102) and 0.593 (95% CI, 0.167–2.100, *p* = 0.418), respectively. Multivariate analysis also showed that the DIO2 Ala/Ala ratio was significantly associated with obesity (Table 3).

### 3.3. Clinical Characteristics of Each SNP Genotype

Table 4 shows the clinical characteristics of DIO2 Thr92Ala (rs225014). BMI, POW, and BMI% in the DIO2 Ala/Ala genotype were significantly higher than those in the DIO2 Thr/Thr genotype (*p* < 0.05). UCP1 SNP, TC, LDLC, and HbA1c levels of the AG genotype were significantly higher than those of the AA genotype (*p* < 0.05). Interestingly, these factors did not differ significantly with the GG genotype (Table 5). β3AR SNP, BMI, POW, and BMI% of the Arg/Arg genotype were significantly lower than those of Trp/Trp (Table 6).

### 3.4. Gender Difference in the Genotype Distribution of SNPs

Genotype distributions of DIO2 Thr92Ala (rs225014), UCP1-3826 A/G (rs1800592), and β3AR Trp64Arg (rs4994) in the NOB and OB groups were analyzed separately for boys and girls. The prevalence of the homozygous Ala allele of DIO2 in the OB group was significantly higher than the NOB group for Thr/Thr and Ala/Ala genotypes in boys (*p* = 0.07) (Table 6) but not in girls (Table 7). Logistic analysis also showed that DIO2 Ala/Ala was associated with obesity, with an OR of 5.917 (95%CI 1.864–18.784, *p* = 0.003) (Table 8). Logistic analysis also showed that DIO2 Ala/Ala was associated with obesity, with an OR of 5.917 (95%CI 1.864–18.784, *p* = 0.003) (Table 9).

## 4. Discussion

Obesity is a highly complex heterogeneous disease caused by multiple factors such as the influence of the environment, lifestyle, and genes. The incidence of obesity in children is increasing worldwide. Pediatric obesity is occurring in younger generations and is becoming more severe compared with previous decades [18]. Obesity is strongly influenced by genetic and environmental factors. Our genes have not changed significantly over the last few decades. Even with melanocortin-4 receptor deficiency, which causes obesity, the rate of obesity differs among generations [19]. In recent years, childhood obesity has increased during the COVID-19 pandemic [20]. The review of this information suggests that modern society is an “obesogenic” environment. However, not everyone will become obese because they are in the same environment. Results from family and twin studies have suggested that genetic factors explain 40% to 70% of the inter-individual variation in obesity susceptibility [2]. Individuals with pediatric obesity frequently remain obese through adulthood. It is important to elucidate the interaction between genetic factors and pediatric obesity and manage obesity in childhood. Recently, BAT has been identified as a novel target for obesity, as it increases energy expenditure by non-shivering thermogenesis. BAT-related gene polymorphisms are associated with obesity [4,5,6,7]. However, these relationships have not yet been fully elucidated, especially in pediatric obesity. Therefore, a case-control study was conducted to investigate the association of pediatric obesity with three SNPs (DIO2 Thr92Ala (rs225014), UCP1-3826 A/G (rs1800592), β3AR Trp64Arg (rs4994)) related to BAT. We demonstrated a significant correlation between DIO2 Thr92Ala (rs225014) polymorphism and childhood obesity. 

In the present case-control study, the frequency of the homozygous Ala allele of DIO2 was significantly higher in the OB than in the NOB group (17.1% vs. 5.8%, *p* = 0.004) (Table 2). These frequencies resulted in an OR of 4.036 (95% CI 1.785–9.124) for the DIO2 Ala/Ala genotype in the OB group (Table 3). More than 40% of subjects with DIO2 Ala/Ala were obese, which was the highest prevalence of obesity among all genotypes. This result is similar to that of a previous study showing that the DIO2 Ala/Ala genotype is strongly associated with obesity, concomitant with insulin resistance in a large cohort of patients with type 2 diabetes mellitus [5]. Individuals with DIO2 Ala/Ala tend to have insulin resistance, with a lower glucose disposal rate among adult obese Caucasians [7]. DIO has three isoforms: DIO1, DIO2, and DIO3. DIO1 and DIO2 convert T4 to T3 by catalyzing 5′-deiodination, and DIO1 and DIO3 convert T4 to an inactive metabolite, rT3, by catalyzing 5-deiodination [21]. DIO2 exhibits a higher catalytic capacity than DIO1 and therefore plays a critical role in the production of T3 [22,23]. TH (T4 and T3) regulates metabolic processes, such as body weight, lipolysis, and thermogenesis [24]. DIO2 Ala mutation impairs DIO2-mediated conversion of T4 to T3 in thyroid-deficient patients [11,25,26,27,28], and DIO2 Ala mutation reduces intracellular conversion of T4 to T3 compared to the DIO2 wild type [27]. Therefore, DIO2 Ala mutation could reduce TH-mediated thermogenesis of BAT, leading to obesity. However, not all studies examining this genotype have verified its association with obesity, despite subjects having insulin resistance [23,29]. A similar discrepancy was observed in this study, which might be due to differences in age. Young individuals aged ≤18 years were enrolled in the present study. In contrast, all previous studies enrolled people aged ≥40 years. BAT mass is high during infancy and declines through adulthood [30]. Therefore, BAT might impact childhood more than adulthood. Furthermore, the activity of BAT increases during childhood, reaches its peak at approximately 12 years, and then declines through adulthood [31]. The average age of children enrolled in the present study was 12 years. Thus, this concordance in age may have affected the results. We hypothesized that adolescents approximately 12 years of age are suitable for assessing the association between obesity and gene polymorphisms related to BAT because other factors, such as environment, lifestyle, and genetic factors, were likely to be more involved in the pathogenesis of obesity during adulthood. Further, insulin resistance may be the main phenotype caused by DIO2 polymorphism in adulthood. DIO2 is also expressed in skeletal muscle, and TH upregulates the expression of glucose transporter 4, which is responsible for glucose uptake. Therefore, skeletal muscle is the main glucose-consuming tissue. DIO2 polymorphism might lead to insulin resistance in adulthood, as skeletal muscle mass is more abundant in adulthood than in children. More importantly, there were no significant differences in the insulin levels between the DIO2 genotypes after adjusting for body weight. The higher insulin levels in the DIO2 Ala/Ala genotype may be secondary to obesity.

Previous studies have demonstrated that DIO2, UCP1, and β3AR have a mutual impact on obesity in adulthood [6,7]. Therefore, the present study examined the gene–gene interaction of the DIO2 Thr92Ala (rs225014) genotype with UCP1-3826 A/G (rs1800592) and β3AR Trp64Arg (rs4994) in pediatric obesity [32]. There was no significant association between DIO2 Thr92Ala (rs225014) and UCP1-3826 A/G (rs1800592) or β3AR Trp64Ar (rs4994) in pediatric obesity. This discrepancy between studies that investigated adult obesity and the present study may also be due to age. The combination of UCP1 and β3AR polymorphisms is related to reduced BAT mass with age [33], suggesting that aging uncovers the effects of this gene combination on obesity. Further studies are needed to investigate the effect of UCP1 and/or the combination of DIO2 and UCP1 on pediatric obesity. Another possibility may be sex-related BAT activity. Females have greater BAT mass than males, and sex differences may be greater in younger generations. Additionally, females can regulate their thermogenesis more than males, as sympathetic innervation in BAT is more abundant in females. However, the effects of these SNP differences remain unknown. Sex-related differences in BAT are of great concern. In fact, the effect of the DIO2 polymorphism differed by sex in the present study. DIO2 Ala/Ala was associated with obesity in boys but not in girls. Further studies are needed to investigate the association between gene polymorphisms related to BAT with aging and sex differences. Some studies have reported that polymorphisms in UCP1 and β3AR have an effect on obesity even in children. This difference from our findings may be related to differences in the ethnicities of the study populations. However, Endo et al. reported an association between β3AR Trp64Arg and obesity in Japanese school children. The difference in the sex ratio in each study may also explain the inconsistency between the results. As this was an exploratory study, further verification of our results is warranted.

This is the first study to report an association between the DIO2 Thr92Ala (rs225014) polymorphism and pediatric obesity. It also suggests that BAT-related SNPs should be assessed in patients with pediatric obesity. However, this study had several limitations. (1) Owing to its exploratory nature, the sample size was not determined statistically, and the multiplicity of tests was not considered during analysis. (2) There was a difference in the mean age between the NOB and OB groups. The mean height is expected to increase with age: the taller the children, the higher their BMIs. (3) There is a lack of data on the pubertal stage. Puberty drastically alters body composition according to sex. During puberty, boys gain only lean mass, whereas girls gain both lean mass and fat mass. Therefore, the effect of altered body composition during puberty could not be assessed due to a lack of data on the pubertal stage. (4) Population-based non-obese children were included as controls in the present study. However, perhaps hospital-based non-obese children should have been enrolled as controls to match the backgrounds of all the participants. (5) We did not have data on thyroid function. DIO2 Thr92Ala (rs225014) might be associated with obesity via hypothyroidism [34]. Due to these limitations, further studies are needed to validate the results.

## 5. Conclusions

In conclusion, the present study indicates that the homozygous Ala/Ala allele of DIO2 is associated with an increased risk of obesity in children. It also suggests that pediatric obesity may be suitable for assessing the manner in which gene polymorphisms are related with BAT. These results indicate that it is important to assess the DIO2 Thr92Ala (rs225014) polymorphism in pediatric obesity, and functional investigations such as infrared thermography are necessary to further elucidate the findings.

## Figures and Tables

**Table 1:** Clinical characteristics classified based on POW, in the non-obese (NOB) and obese (OB) children groups.

	NOB (n = 274)	OB (n = 82)	*p*
POW (%)	−4.4 (−11.2–3.2)	46.4 (36.8–57.2)	<0.001
Age (years)	13.5 (12.9–14.1)	11.1 (9.0–13.0)	<0.001
Gender male/ female	141/133	47/35	0.379
Height (cm)	155.7 (150.5–160.8)	146.1 (134.0–157.5)	<0.001
Body weight (kg)	45.2 (40.4–50.8)	58.8 (45.2–68.0)	<0.001
BMI (kg/m^2^)	18.6 (17.3–19.8)	25.9 (24.1–28.1)	<0.001
BMI %ile	39.5 (17.7–58.9)	98.0 (95.9–99.1)	<0.001
TC (mg/dL)	169.0 (152.0–186.0)	^a^ 176.0 (161.0–198.0)	0.010
HDLC (mg/dL)	66.0 (58.0–75.0)	^a^ 52.0 (43.0–61.0)	<0.001
LDLC (mg/dL)	88.0 (73.0–106.0)	^a^ 106.0 (88.0–123.0)	<0.001
nonHDLC (mg/dL)	101.5 (85.0–120.0)	^a^ 127.0 (106.0–144.0)	<0.001
HbA1c (%)	5.2 (5.1–5.3)	^b^ 5.5 (5.2–5.6)	<0.001

Data are presented as medians (25th–75th) and n. NOB, non-obese children group; OB, obese children group; BMI, body mass index; POW, percentage of overweight; TC, total cholesterol; HDLC, high-density lipoprotein cholesterol; LDLC, low-density lipoprotein cholesterol; HbA1c, hemoglobin A1c. ^a^ n = 81, ^b^ n = 76.

**Table 2:** Genotype distributions of SNPs.

	NOB (n = 274)	OB (n = 82)	*p*
DIO2 Thr92Ala			0.004
(rs225014)			
Thr/Thr	0.522 (143)	0.378 (31)	
Thr/Ala	0.420 (115)	0.451 (37)	
Ala/Ala	0.058 (16)	0.171 (14) *	
UCP1 −3826 A/G			0.123
(rs1800592)			
AA	0.350 (96)	0.232 (19)	
AG	0.398 (109)	0.476 (39)	
GG	0.252 (69)	0.293 (24)	
*β*3ADR Trp64Arg			0.716
(rs4994)			
Trp/Trp	0.650 (178)	0.646 (53)	
Trp/Arg	0.288 (79)	0.317 (26)	
Arg/Arg	0.062 (17)	0.037 (3)	

Numbers in the parentheses indicate the number of the subjects. * Compared to the NOB group for the Thr/Thr and Ala/Ala genotypes (*p* = 0.004).

**Table 3:** Uni- and multivariate analyses with respect to obesity classified based on the percentage of weight.

		Univariate Analysis					Multivariate Analysis (Full Model)					Multivariate Analysis (Stepwise)			
	ß	*p*-Value	Odds	95%CI		ß	*p*-Value	Odds	95%CI		ß	*p*-Value	Odds	95%CI	
DIO2 Thr92Ala		0.003					0.007					0.003			
Thr/Thr			ref					ref					ref		
Thr/Ala	0.395	0.149	1.484	0.868	2.538	0.289	0.305	1.335	0.768	2.321	0.395	0.149	1.484	0.868	2.538
Ala/Ala	1.395	0.001	4.036	1.785	9.124	1.323	0.002	3.757	1.645	8.580	1.395	0.001	4.036	1.785	9.124
UCP1−3826 A/G		0.135					0.258								
AA			ref					ref							
AG	0.592	0.058	1.808	0.979	3.338	0.517	0.110	1.678	0.890	3.162					
GG	0.564	0.102	1.757	0.893	3.458	0.450	0.207	1.568	0.779	3.157					
β3ADR Trp64Arg		0.642					0.848								
Trp/Trp			ref					ref							
Trp/Arg	0.100	0.716	1.105	0.645	1.895	0.020	0.944	1.020	0.583	1.784					
Arg/Arg	−0.523	0.418	0.593	0.167	2.100	−0.367	0.579	0.693	0.190	2.531					

**Table 4:** Clinical characteristics of the DIO2 Thr92Ala (rs225014) SNP genotype.

	DIO2 Thr/92Ala (rs225014)		
	Thr/Thr (n = 174)	Thr/Ala (n = 152)	Ala/Ala (n = 30)
Age (years)	13.6 (12.9–14.2)	13.1 (12.4–13.9) **	12.9 (11.1–13.9)
Gender male/ female	89/ 85	84/ 68	15/ 15
Body height (cm)	155.4 (150.2–160.9)	154.5 (146.1–160.2)	151.4 (142.0–159.5) *
Body weight (kg)	46.5 (40.6–52.8)	46.1 (40.4–52.2)	49.7 (42.1–56.0)
BMI (kg/m^2^)	19.1 (17.5–20.8)	19.1 (17.7–22.7)	21.8 (19.3–26.3) **
POW (%)	0.2 (−9.6–10.9)	−0.5 (−9.0–24.9)	16.1 (−3.3–46.4) **
BMI %ile	48.5 (20.2–72.7)	51.9 (27.4–87.5)	75.2 (43.9–97.9) **
TC (mg/dL)	169.0 (152.0–186.0)	^b^ 170.0 (154.0–192.0)	176.0 (163.0–193.0)
HDLC (mg/dL)	64.0 (55.0–73.0)	^b^ 63.0 (54.0–73.0)	62.0 (52.0–68.0)
LDLC (mg/dL)	89.5 (73.0–108.0)	^b^ 93.0 (78.2–112.0)	96.5 (84.0–109.0)
nonHDLC (mg/dL)	103.5 (84.0–124.0)	^b^ 108.0 (90.0–126.0)	110.5 (100.0–127.0)
HbA1c (%)	^a^ 5.2 (5.1–5.4)	^c^ 5.2 (5.1–5.4)	^d^ 5.2 (5.0–5.5)

Data are presented as median (25th–75th) and n. BMI, body mass index; POW, percentage of overweight; TC, total cholesterol; HDLC, high-density lipoprotein cholesterol; LDLC, low-density lipoprotein cholesterol; HbA1c, hemoglobin A1c. * *p* < 0.05 vs. wild type of the corresponding gene, ** *p* < 0.01 vs. wild type of the corresponding gene. ^a^ n = 173, ^b^ n = 151, ^c^ n = 149, ^d^ n = 28.

**Table 5:** Clinical characteristics of the UCP1-3826 A/G (rs1800592) SNP genotype.

	UCP1−3826 A/G (rs1800592)		
	AA (n = 115)	AG (n = 148)	GG (n = 93)
Age (years)	13.4 (12.9–14.1)	13.1 (12.6–13.9) *	13.3 (12.5–14.2)
Gender male/ female	55/60	80/68	53/40
Body height (cm)	155.0 (149.7–159.6)	154.7 (147.5–160.5)	155.7 (147.1–160.3)
Body weight (kg)	45.3 (40.0–52.2)	46.4 (41.5–52.8)	48.4 (42.0–53.4)
BMI (kg/m^2^)	19.1 (17.4–20.9)	19.3 (17.7–23.0)	19.4 (17.8–24.1)
POW (%)	0.0 (−9.9–11.6)	1.2 (−8.8–20.7)	1.4 (−8.8–26.0)
BMI %tile	43.9 (18.9–73.2)	51.7 (28.5–88.8)	53.8 (28.3–89.0)
TC (mg/dL)	165.0 (148.0–186.0)	173.0 (157.0–191.5) *	^c^ 170.0 (153.0–189.5)
HDLC (mg/dL)	64.0 (56.0–71.0)	64.0 (55.0–72.5)	^c^ 61.0 (49.5–74.0)
LDLC (mg/dL)	87.0 (72.0–105.0)	94.0 (79.0–113.0) *	^c^ 91.5 (79.5–109.0)
nonHDLC (mg/dL)	100.0 (83.0–122.0)	111.0 (91.5–128.5) *	^c^ 107.5 (91.5–125.0)
HbA1c (%)	^a^ 5.2 (5.1–5.3)	^b^ 5.2 (5.1–5.4) **	^d^ 5.3 (5.1–5.4)

Data are presented as median (25th–75th) and n. BMI, body mass index; POW, percentage of overweight; TC, total cholesterol; HDLC, high-density lipoprotein cholesterol; LDLC, low-density lipoprotein cholesterol; HbA1c, hemoglobin A1c. * *p* < 0.05 vs. the wild type of the corresponding gene, ** *p* < 0.01 vs. the wild type of the corresponding gene. ^a^ n = 114, ^b^ n = 145, ^c^ n = 92, ^d^ n = 91.

**Table 6:** Clinical characteristics of the β3ADR Trp64Arg (rs4994) SNP genotype.

	*β*3ADR Trp64Arg (rs4994)		
	Trp/Trp (n = 231)	Trp/Arg (n = 105)	Arg/Arg (n = 20)
Age (years)	13.2 (12.6–13.9)	13.5 (12.7–14.4)	13.4 (12.8–13.8)
Gender male/female	126/105	53/52	9/11
Body height (cm)	154.9 (148.0–160.4)	155.4 (149.7–160.4)	154.2 (150.1–157.6)
Body weight (kg)	46.2 (40.6–53.0)	47.5 (41.6–54.5)	42.0 (38.0–47.7)
BMI (kg/m^2^)	19.2 (17.7–22.2)	19.6 (17.7–23.2)	17.5 (16.2–20.0) *
POW (%)	−0.4 (−8.8–17.0)	2.4 (−8.3–17.8)	−7.3 (−17.3–5.7) *
BMI %ile	51.6 (26.5–86.3)	55.8 (24.5–87.1)	30.3 (7.4–61.2) *
TC (mg/dL)	^a^ 172.0 (153.0–188.0)	168.0 (151.0–190.0)	174.0 (152.0–191.5)
HDLC (mg/dL)	^a^ 63.0 (54.0–72.0)	64.0 (53.0–73.0)	66.5 (57.0–73.0)
LDLC (mg/dL)	^a^ 92.5 (76.0–110.0)	89.0 (76.0–107.0)	88.5 (77.5–110.5)
nonHDLC (mg/dL)	^a^ 106.0 (89.0–126.0)	104.0 (89.0–124.0)	114.5 (86.5–123.5)
HbA1c (%)	^b^ 5.2 (5.1–5.4)	^c^ 5.2 (5.1–5.4)	^d^ 5.2 (5.1–5.3)

Data are presented as median (25th–75th) and n. BMI, body mass index; POW, percentage of overweight; TC, total cholesterol; HDLC, high-density lipoprotein cholesterol; LDLC, low-density lipoprotein cholesterol; HbA1c, hemoglobin A1c. * *p* < 0.05 vs. the wild type of the corresponding gene. ^a^ n = 230, ^b^ n = 228, ^c^ n = 103, ^d^ n = 19.

**Table 7:** Genotype distribution of SNPs in boys.

	NOB (n = 141)	OB (n = 47)	*p*
DIO2 Thr92Ala			0.007
(rs225014)			
Thr/Thr	0.504 (71)	0.383 (18)	
Thr/Ala	0.454 (64)	0.426 (20)	
Ala/Ala	0.043 (6)	0.191 (9) *	
UCP1 -3826 A/G			0.182
(rs1800592)			
AA	0.326 (46)	0.191 (9)	
AG	0.397 (56)	0.511 (24)	
GG	0.277 (39)	0.298 (14)	
*β*3ADR Trp64Arg			1.000
(rs4994)			
Trp/Trp	0.667 (94)	0.681 (32)	
Trp/Arg	0.284 (40)	0.277 (13)	
Arg/Arg	0.050 (7)	0.043 (2)	

The numbers in the parentheses indicate the number of subjects. * Compared to the NOB group on the Thr/Thr and Ala/Ala genotype (*p* = 0.007).

**Table 8:** Genotype distribution of SNPs in girls.

	NOB (n = 133)	OB (n = 35)	*p*
DIO2 Thr92Ala			0.140
(rs225014)			
Thr/Thr	0.541 (72)	0.371 (13)	
Thr/Ala	0.383 (51)	0.486 (17)	
Ala/Ala	0.075 (10)	0.143 (5)	
UCP1 −3826 A/G			0.544
(rs1800592)			
AA	0.376 (50)	0.286 (10)	
AG	0.398 (53)	0.429 (15)	
GG	0.226 (30)	0.286 (10)	
*β*3ADR Trp64Arg			0.546
(rs4994)			
Trp/Trp	0.632 (84)	0.600 (21)	
Trp/Arg	0.293 (39)	0.371 (13)	
Arg/Arg	0.075 (10)	0.029 (1)	

The numbers in the parentheses indicate the number of subjects.

**Table 9:** Univariate and multivariate analyses with respect to obesity classified based on the percentage of weight.

	Univariate Analysis									
		Boy					Girl			
	ß	*p*-Value	Odds	95%CI		ß	*p*-Value	Odds	95%CI	
DIO2 Thr92Ala		0.010					0.164			
(rs225014)										
Thr/Thr			ref					ref		
Thr/Ala	0.209	0.570	1.233	0.599	2.535	0.613	0.136	1.846	0.824	4.135
Ala/Ala	1.778	0.003	5.917	1.864	18.784	1.019	0.103	2.769	0.813	9.429
UCP1−3826 A/G		0.199					0.576			
(rs1800592)										
AA			ref					ref		
AG	0.784	0.074	2.19	0.927	5.175	0.347	0.444	1.415	0.582	3.441
GG	0.607	0.206	1.835	0.717	4.696	0.511	0.310	1.667	0.621	4.470
β3ADR Trp64Arg		0.973					0.490			
(rs4994)										
Trp/Trp			ref					ref		
Trp/Arg	−0.046	0.903	0.955	0.454	2.008	0.288	0.475	1.333	0.606	2.935
Arg/Arg	−0.175	0.832	0.839	0.166	4.249	−0.916	0.395	0.839	0.048	3.301

## Data Availability

The datasets used and/or analyzed during the current study will be made available by the corresponding author upon request.
